# Preexisting cell state rather than stochastic noise confers high or low infection susceptibility of human lung epithelial cells to adenovirus

**DOI:** 10.1128/msphere.00454-24

**Published:** 2024-09-24

**Authors:** Anthony Petkidis, Maarit Suomalainen, Vardan Andriasyan, Abhyudai Singh, Urs F. Greber

**Affiliations:** 1 Department of Molecular Life Sciences, Universitat Zurich, Zurich, Switzerland; 2 Department of Electrical and Computer Engineering, University of Delaware, Newark, Delaware, USA; University of Michigan, Ann Arbor, Michigan, USA

**Keywords:** adenovirus infection, viral gene expression, cell state, infection variability, infection heterogeneity, noise, fluorescence microscopy, early viral gene expression, DNA virus

## Abstract

Viruses display large variability across all stages of their life cycle, including entry, gene expression, replication, assembly, and egress. We previously reported that the immediate early adenovirus (AdV) E1A transcripts accumulate in human lung epithelial A549 cancer cells with high variability, mostly independent of the number of incoming viral genomes, but somewhat correlated to the cell cycle state at the time of inoculation. Here, we leveraged the classical Luria–Delbrück fluctuation analysis to address whether infection variability primarily arises from the cell state or stochastic noise. The E1A expression was measured by the expression of green fluorescent protein (GFP) from the endogenous E1A promoter in AdV-C5_E1A-FS2A-GFP and found to be highly correlated with the viral plaque formation, indicating reliability of the reporter virus. As an ensemble, randomly picked clonal A549 cell isolates displayed significantly higher coefficients of variation in the E1A expression than technical noise, indicating a phenotypic variability larger than noise. The underlying cell state determining infection variability was maintained for at least 9 weeks of cell cultivation. Our results indicate that preexisting cell states tune adenovirus infection in favor of the cell or the virus. These findings have implications for antiviral strategies and gene therapy applications.

**IMPORTANCE:**

Viral infections are known for their variability. Underlying mechanisms are still incompletely understood but have been associated with particular cell states, for example, the eukaryotic cell division cycle in DNA virus infections. A cell state is the collective of biochemical, morphological, and contextual features owing to particular conditions or at random. It affects how intrinsic or extrinsic cues trigger a response, such as cell division or anti-viral state. Here, we provide evidence that cell states with a built-in memory confer high or low susceptibility of clonal human epithelial cells to adenovirus infection. Results are reminiscent of the Luria–Delbrück fluctuation test with bacteriophage infections back in 1943, which demonstrated that mutations, in the absence of selective pressure prior to infection, cause infection resistance rather than being a consequence of infection. Our findings of dynamic cell states conferring adenovirus infection susceptibility uncover new challenges for the prediction and treatment of viral infections.

## INTRODUCTION

Biological systems and human susceptibility to virus infections are inherently variable due to genetic and environmental factors, as well as the complex stochastic nature of biochemical reactions and interactions [for reviews, see (1–4)]. Although biological systems follow well-defined physical laws at the molecular level, the sheer number of their reactions precludes deterministic modeling at relevant spatiotemporal scales (1, 5). This difficulty cannot be easily overcome by simplifying assumptions about the underlying processes, and meaningful models addressing the heterogeneity within biological systems are difficult to establish (5). Yet, on a macroscopic level, cells display a seemingly deterministic behavior, which they achieve by complex regulatory networks involving feedback loops, checkpoints, redundancy, and noise-attenuating mechanisms (6, 7).

Isogenic populations of cells are known to vary in their phenotypes and gene expression patterns (8), a phenomenon classically assigned to intrinsic and extrinsic noise, where noise is operationally defined as the standard deviation divided by the mean of a measurement. Explaining phenotypic variability down to the molecular level is a significant challenge, especially when aiming at causality beyond correlation. Conceptually, one can distinguish between “intrinsic” noise caused by stochastic processes, such as transcription factors binding to the interferon (IFN) beta promoter sequence (9), and “extrinsic” noise caused by cellular heterogeneity, such as the metabolic, cell cycle, or signaling state of a cell, with the latter likely containing a deterministic component (1, 10). In the case of gene expression, the distinction between intrinsic and extrinsic noises can, for example, be made by experimentally comparing the expressions of two genes governed by the same promoters within single cells using fluorescent reporters (11, 12) or, more recently, by high-throughput single-cell technologies probing the accessibility of the chromatin in the promoter sequence using assay for transposase-accessible chromatin using sequencing (ATAC-seq) (13). Although binding of transcription factors and RNA polymerase II (Pol-II) to open chromatin at promoters, followed by transcription initiation and elongation, are subject to stochasticity, deterministic factors, including the promoter sequence, promoter–enhancer interactions, and epigenetic modifications also contribute to variability in gene expression (14, 15). Deterministic factors are also derived from the overall three-dimensional genome organization with non-random positioning of chromosomes and genes in the cell nucleus, distinct chromatin compartments, and topologically associating domains (16).

Cellular variability and stochastic processes of viral infections are critical to overall infection dynamics, as exemplified by stochastic variations of transcription and viral burst size (17–20), and modeling approaches (21–23). Notwithstanding, infection variability is highly complex and determined by both viral and host factors and their interactions (23). Adenoviruses (AdVs), for example, exhibit large cell-to-cell infection variability throughout all stages of their life cycle, including entry, genome delivery, transcription, assembly, and egress (4, 18, 24–26). Human AdVs elicit respiratory, gastrointestinal, and eye diseases and persist in lymphoid cells of the mucosal tissue (27, 28). In immunosuppressed individuals, large titers of AdV disseminate and lead to life-threatening conditions. *In vitro* studies with normal human fibroblasts have shown that AdV infection is suppressed by type 1 or 2 IFN, which restricts the expression of the immediate early viral gene E1A by recruiting E2F-mediated restriction factors to the E1A enhancer/promoter (29, 30). E1A is the major AdV transactivator promoting the expression of other viral promoters (27, 31, 32). It plays a pivotal role in driving the cell to the S-phase of the cell cycle, thereby boosting viral replication and also suppressing cellular immune response and differentiation (33, 34). We previously described a large variability of E1A transcription from incoming viral DNAs visualized by 5-ethynyl-2′-deoxycytidine (EdC)-tagging and copper(I)-catalyzed azide–alkyne cycloaddition (click) reaction (35). E1A transcripts, as observed by single-molecule mRNA fluorescence *in situ* hybridization (smFISH), were particularly enhanced in cells in the G1 cell cycle phase, although transcriptional activity remained highly variable between different cells and viral genomes throughout other phases of the cell cycle, indicating that the cell cycle state is not the sole determinant for E1A expression variability.

Here, we address the question of whether E1A transcriptional variability in AdV-infected isogenic cultured cells arises primarily from stochasticity or distinct cell states. Leveraging the classical Luria and Delbrück (36) fluctuation test for reversible switching between transient cellular states (37), our results show that AdV infections determined by immediate early E1A expression exhibit high or low efficiency depending on cell states with a built-in memory longer than 9 weeks. The E1A expression was found to be tightly correlated with viral plaque formation, indicating that distinct cell states impact the virus titer formation.

## RESULTS

### Isolation of clonal populations of A549 cells and experimental setup

**Fig 1:**
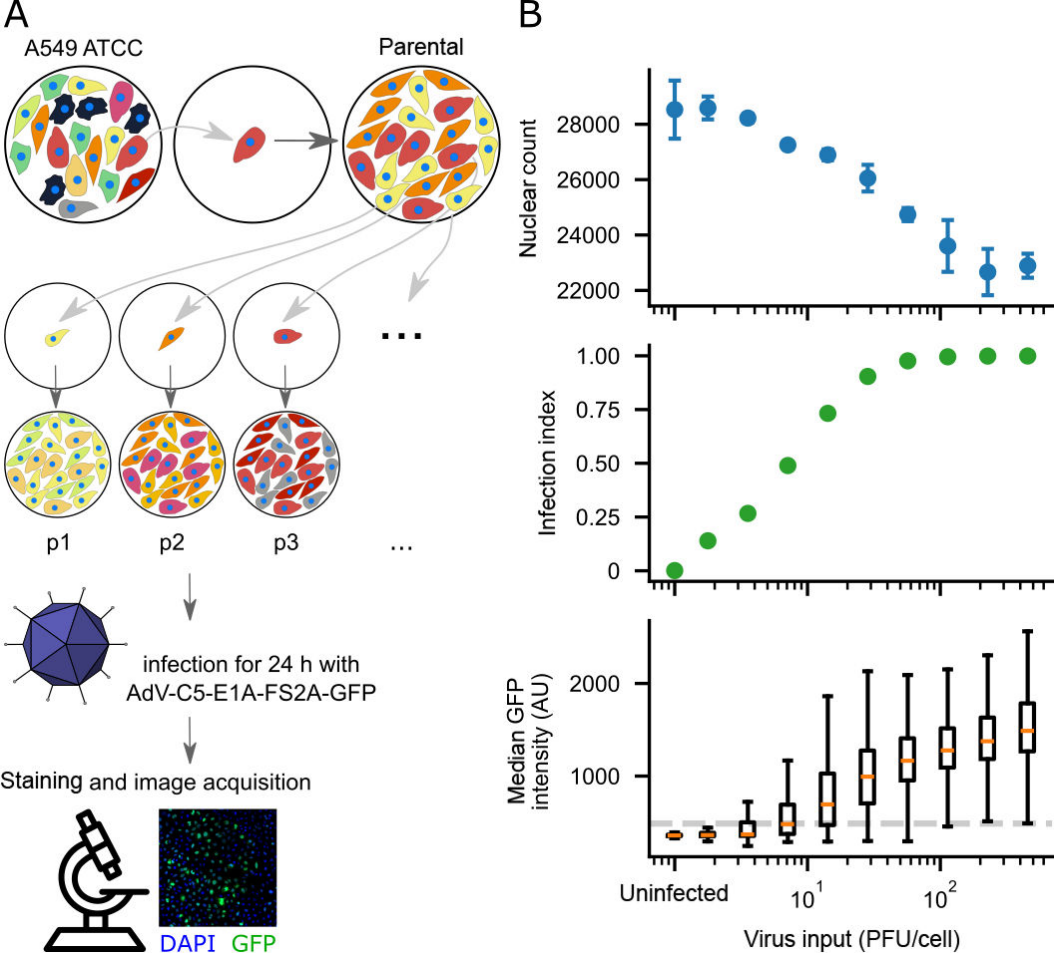
Experimental setup and determination of virus concentration. (**A**) Isolation of clonal cell populations and workflow of the infection experiment. To grow clonal cell populations, individual A549 cancer cells were isolated from a cell culture by limiting dilution and grown to form colonies (“parental” populations, one is shown). Subsequently, from five different “parental” populations, up to 40 “subpopulations” were derived (three shown). Colors symbolize cellular variability, which decreases from the starting culture (A549) to the subpopulations. For the infection experiment, cells were seeded overnight in imaging plates and infected on the next day by adding AdV-C5-E1A-FS2A-GFP, which produces green fluorescent protein (GFP) under control of the E1A promoter. The infection was left to progress for 24 h when cells were fixed, permeabilized, stained with 4′,6-diamidino-2-phenylindole (DAPI), and imaged at a high-throughput fluorescence microscope. (**B**) Titration of AdV-C5-E1A-FS2A-GFP on A549 cells. The calculation of virus input in plaque-forming units (PFUs) per cell is performed under the assumption that cells have doubled overnight after seeding. For subsequent infection experiments, a virus concentration was chosen that resulted in an infection index of 50%. The dotted line in the bottom panel shows the threshold for the infection cutoff, which was determined as the 99.9th percentile of the median GFP intensity of the uninfected control populations. For the first two plots, error bars indicate standard deviations, *n* = 3. In the boxplot, the line indicates the median; the boxes show the quartile ranges; and the whiskers extend to 1.5 times the interquartile range (IQR). The *x*-axis position of the uninfected condition is chosen arbitrarily, as 0 µl could not be placed on a logarithmic axis.

As previous reports demonstrated that cancer cell lines display considerable genetic heterogeneity (38–40), we started our investigation by isolating single cells and growing them in clonal founding populations. To this end, A549 lung epithelial cancer cells were trypsinized, passed through a cell strainer to remove clumped cells, and seeded at a density of one cell per well by limiting dilution. Transmitted light microscopy images were acquired regularly to ensure that the colonies indeed originated from single cells and to monitor cell growth. Once these “parental” A549 cell populations were obtained, the procedure was repeated to create up to 40 clonal “subpopulations” from a given parental population (Fig. 1A). The subpopulations were expanded, seeded in imaging well plates overnight, and infected continuously for 24 h with AdV-C5-E1A-FS2A-GFP, which expresses green fluorescent protein (GFP) under control of the native viral E1A promoter (41, 42). Cells were fixed, permeabilized, stained with 4′,6-diamidino-2-phenylindole (DAPI), and imaged by fluorescence microscopy in an automated high-throughput microscope (ImageXpress Micro Confocal IXM-C, Molecular Devices). The DAPI signal was used for nuclear segmentation, and the infection state was assessed based on the median nuclear GFP intensity. The virus concentration was chosen such that the amount of input virus resulted in an A549 cell infection index of 50%, allowing the detection of changes in infection susceptibility in an optimal dynamic range (Fig. 1B). The infection cutoff was determined as the 99.9th percentile of the median nuclear GFP intensity of the uninfected control, which resulted in a robust threshold (Fig. S1). Notably, the nuclear size, which is known to be increased in the G2-phase compared to G0-, G1-, and S-phases of the cell cycle (35, 43), had only a small effect on the infection index (Fig. S2A). This suggests that cell cycle-dependent effects are comparatively minor, which is in line with previous findings (35).

### Cellular states confer early and late infection variability

**Fig 2:**
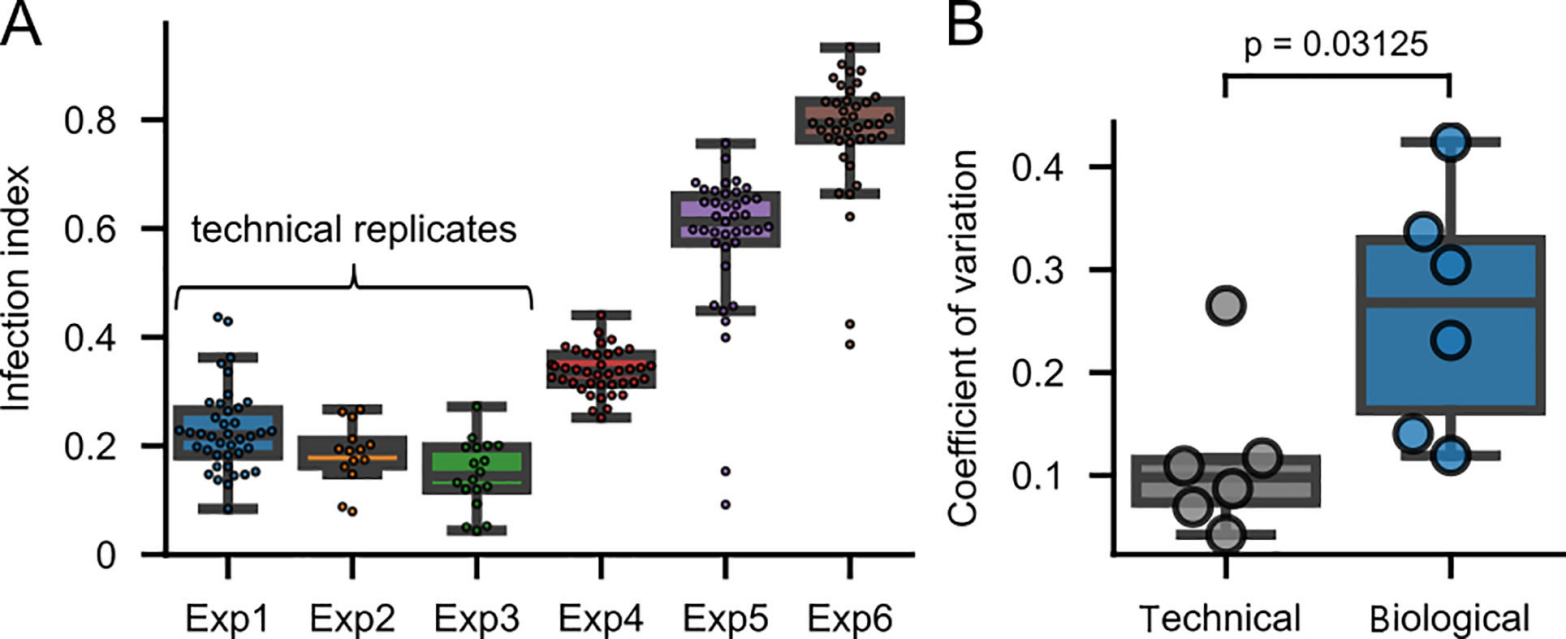
Biological and technical variabilities in infection of clonal populations. (**A**) Infection indices across clonal populations in six independent experiments. Populations were derived either from the same parental population and were therefore technical replicates (experiments 1–3) or from different parental populations (experiments 4–6). The line indicates the median; the boxes show the quartile ranges; and the whiskers extend to 1.5 times the interquartile range (IQR). Data points are overlaid. Exp 1: *n* = 40, Exp 2: *n* = 14, Exp 3: *n* = 18, Exp 4: *n* = 40, Exp 5: *n* = 39, and Exp 6: *n* = 40. (**B**) Comparison of technical and biological noise sources. The coefficient of variation (CV) is computed for the six experiments shown in (**A**). The technical CV was calculated as $\frac{1}{n}\sum_{i=1}^{n}\frac{\sigma_{i}}{\mu_{i}}$, and the biological CV as $\frac{\sigma }{\frac{1}{n}\sum_{i=1}^{n}\mu_{i}}$, where Σ is the sum operator; n is the number of samples; σ is the standard deviation; μ is the mean; and i refers to the index of a sample. For calculation of the technical CV, $\sigma_{i}$ refers to the standard deviation of the technical replicates of a given biological sample, while for the biological CV, σ refers to the standard deviation across all means of all biological samples. The *P* value indicates the result of a two-sided, non-parametric Wilcoxon signed-rank test performed in SciPy (44), *n* = 6.

To determine the infection variability in clonal cell populations, we infected populations derived from four different parental populations in six independent experiments as described above. The populations showed considerable deviations in the mean and distribution of their infection indices (Fig. 2A). Next, we compared technical and biological sources of noise (variability) across all experiments. We defined technical noise as the average coefficient of variation (CV) across different technical replicates of the same population and biological variability as the average CV across different populations. Our results indicate that the biological variability (mean CV 0.259) was significantly larger than technical noise (mean CV 0.115) (Fig. 2B). This indicates a strong contribution of an at least transiently heritable factor impacting the cellular state and the permissiveness to early viral gene expression.

To probe whether differential infection susceptibility is observed at stages subsequent to early infection, we used the reporter virus AdV-C5-IX-FS2A-GFP (41), which expresses GFP under control of the viral protein IX promoter, which is active at intermediate time points, that is, after the initiation of early gene expression and before late gene expression (45). Clonal populations were infected either with AdV-C5-E1-FS2A-GFP and assessed for their infection index at 1 dpi or infected with AdV-C5-IX-FS2A-GFP and assessed for the number of plaques at 4 dpi. Cell populations showed a high Spearman R correlation of 0.74 between early and late infection susceptibilities (Fig. S2B). This suggests that differential infection susceptibility is robust and manifested at both early and intermediate times.

### Cellular memory is retained for at least 4 weeks

**Fig 3:**
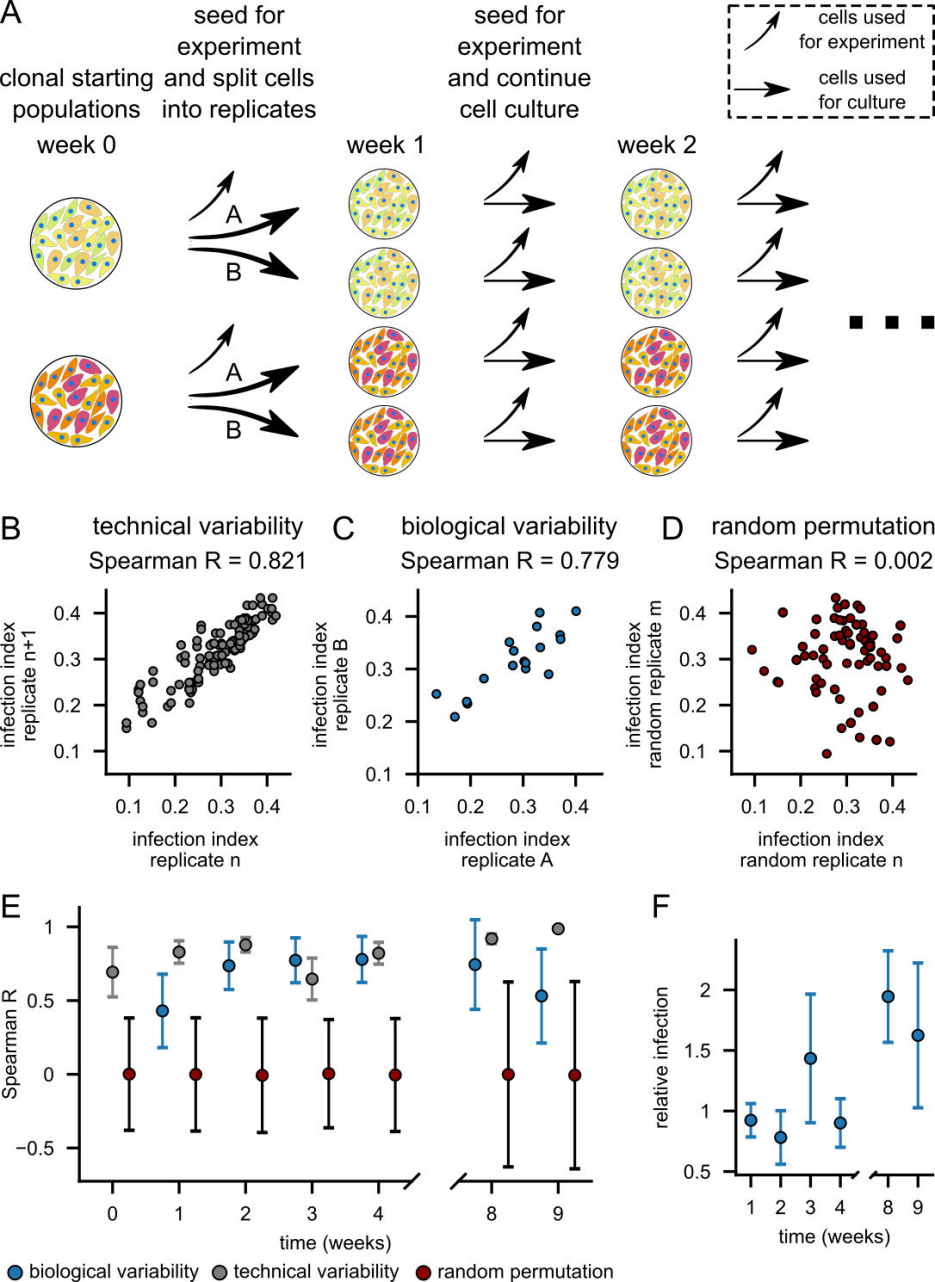
Cellular memory is retained for at least 9 weeks. (**A**) Workflow of the memory experiment. Eighteen selected subpopulations (two are shown) previously derived from one parental population were split into two biological replicates. The resulting 36 cultures were propagated for 9 weeks, and their infection susceptibility was assessed regularly. (**B**) Example of technical correlation between individual technical replicates after 4 weeks, *n* = 108. (**C**) Example of biological correlation between biological replicates A and B after 4 weeks, *n* = 18. (**D**) Example of baseline correlation between randomly chosen populations, *n* = 108. (**E**) Development of correlation coefficients over time. Technical, biological, and random permutation correlation coefficients were calculated as shown in (**B**), (**C**), and (**D**), respectively. Error bars indicate 95% confidence intervals. Week 0 refers to the timepoint of splitting the cells into biological replicates; hence, the biological correlation could only be determined from week 1. Biological correlation: *n* = 18. Technical and random correlation: *n* = 108. (**F**) Trends in temporal susceptibility changes across populations normalized to the infection index of A549 cells at the respective timepoints. The line shows means, and the shaded area shows standard deviation, *n* = 36.

Next, we wondered if the cellular state giving rise to differential infection susceptibility is a stable phenomenon. To address this question, we selected 18 different populations derived from the same parental population and divided each of them into two replicates, A and B. The replicates were cultured and assessed for their infection susceptibility every week over the course of 4 weeks (Fig. 3A). In the absence of transcriptional memory, biological replicates would be expected to show no correlation in their infection indices (46), while in the case of strong memory, biological replicates would exhibit correlation similar to the technical variability. Technical variability was calculated by correlating the individual technical replicates (Fig. 3B), and biological variability by correlating the populations A and B (Fig. 3C). For the baseline correlation, a random permutation of the sample labels was performed, which resulted in a correlation coefficient of around 0 (Fig. 3D). The biological correlation remained high over the course of the experiment, indicating that population-specific infection susceptibility is a stable phenomenon lasting at least for 9 weeks (Fig. 3E). We observed that moderate fluctuations of susceptibility compared to A549 cells occurred over time, but the overall infection susceptibility remained stable (Fig. 3F).

## DISCUSSION

Heterogeneity is an inherent feature of biological systems. For example, population variability has been observed with drug-resistant cancer cell colonies derived from single-cell clones (47, 48), with clonal human lymphocytes exhibiting long-term, heritable differences in gene expression coinciding with clonal variability in thousands of regulatory genomic regions (49), as well as infectious agents, including reactivation of human immunodeficiency virus from latency (11, 50, 51). Clonal infection variability at the phenotypic level has been attributed to genetic and non-genetic factors of virus and host, as well as stochasticity, so-called noise. The AdV infection heterogeneity of isogenic cells has been observed phenotypically at the single-cell level and visualized by fluorescence microscopy of viral reporter genes in live cell plaque assays (18, 52–54).

Here, we found that the clonal cell variability in immediate early AdV E1A expression was significantly larger than pure technical noise. For infection assays, we used the reporter virus AdV-C5-E1A-FS2A-GFP, where the cotranslational ribosomal skipping activity of the 2A peptide gives rise to isometric levels of GFP and E1A (41). Besides transcriptional E1A variability previously observed with smFISH assays (35), the GFP readout entails additional variability at post-transcriptional levels, including mRNA processing, stability, or translation (41). Furthermore, noise-attenuating or -amplifying effects have been ascribed to the steps of mRNA nuclear export (55, 56) and translation (57, 58). Unfortunately, a conclusive comparison of AdV-C5-E1A-FS2A-GFP and AdV-C5 E1A early gene expression was not feasible due to a rather modest correlation of 0.48 for GFP fluorescence and immunostained E1A (Fig. S2C). Underlying reasons for the low correlation may be the different detection limits for GFP and E1A, differences in protein folding kinetics between GFP and E1A, and variable accessibility of the E1A epitope for the M58 monoclonal antibody. Another possible reason for the low correlation of AdV-C5-E1A-FS2A-GFP and AdV-C5 E1A early gene expression may be the rather low ribosomal skipping efficiency of E1A-FS2A-GFP, reaching about 50% (41).

Mechanistically, noise arises from cellular components that undergo intrinsic fluctuations in their concentrations and thereby impart extrinsic noise for their interacting components, which, in this case, is viral early gene transcription. Comparison of the coefficients of variation between biological and technical replicates suggested a large contribution of cell state-dependent factors. The nature of the underlying cellular states, however, remains elusive. An additional open question has been how cell state variability arises and what determines the duration of the low and high infection susceptibility state. Conceivably, this may relate to viral persistence, a widespread phenomenon across human infections with DNA and RNA viruses (59). Interestingly, research in microbiology has demonstrated that bacterial cultures may survive antibiotic treatment due to bacterial persistence or tolerance, notably without bearing genetic resistance (60). Persistence may result from non-genetic single-cell heterogeneity and can arise or disappear spontaneously (61). While this could occur in a probabilistic manner, several deterministic models would also offer plausible explanations, which may involve molecular oscillators, genetic circuits, and sensing of environmental cues (8, 62–64).

Employing high-throughput molecular biology techniques, including RNA sequencing and proteomics, may help to unveil correlations between molecular phenotypic variability and changes in the cell state that impact viral gene expression. Such cell state-dependent variability, rather than low signal fidelity due to stochastic noise, was suggested to be the main driving factor for differential cellular response to interferon-γ stimulation (65). Conceivably, pre-existing differences in cell activity, akin to preexisting mutations in the classical Luria–Delbrück fluctuation test (36), can be amplified by infection and lead to different outcomes. Furthermore, the exploration of environmental factors, such as a population context (66), may provide insight into features that determine the cellular state. Shedding more light on virus–pathogen interactions may also be aided by developing comprehensive computational models that integrate stochastic and deterministic factors and simulate infection outcomes. In conclusion, our results extend the classical Luria–Delbrück (36) fluctuation test and imply reversible switching between transient cellular states, as observed with bacterial cell lineages (37).

## MATERIALS AND METHODS

### Experimental model and subject details

#### Cell culture

Cell lines were cultivated in Dulbecco’s Modified Eagle Medium (DMEM, D6429; Sigma-Aldrich, St. Louis, USA) supplemented with 10% fetal bovine serum (FBS, 10270-106; Gibco, Carlsbad, USA) and non-essential amino acids (M7145; Sigma-Aldrich, St. Louis, USA). Cells were incubated in an environment of 37°C, 5% CO_2_, and 95% humidity. All cultures were passaged when they reached confluency by washing with PBS and trypsinization (C-41020; Trypsin–EDTA, Sigma-Aldrich, St. Louis, USA).

#### Viruses

AdV-C5 and AdV-C5-E1A-FS2A-GFP were kindly provided by Silvio Hemmi (University of Zurich, Switzerland). AdV-C5-E1A-FS2A-GFP was constructed as previously described (42) by inserting an enhanced GFP cassette along with a furin cleavage site (FS) and a ribosome-skipping 2A sequence derived from the foot and mouth disease virus (67–69) after the E1A gene (41). This leads to the expression of GFP under the control of the E1A promoter. See Table 1.

**TABLE 1:** Key ressources

Virus strains		
Adenovirus C5	Kindly provided by Silvio Hemmi (University of Zurich, Switzerland)	
Adenovirus C5-E1A-FS2A-GFP	Kindly provided by Silvio Hemmi (University of Zurich, Switzerland) (42)	
Adenovirus C5-IX-FS2A-GFP	Kindly provided by Silvio Hemmi (University of Zurich, Switzerland) (41)	
Cell culture reagents		
DMEM medium	Sigma-Aldrich	Cat #D6429
Non-essential amino acids (NEAA)	Sigma-Aldrich	Cat #M7145
Fetal bovine serum (FBS)	Gibco	Cat #10270–106
Penicillin–streptomycin	Sigma-Aldrich	Cat #P0781
Trypsin–EDTA	Sigma-Aldrich	Cat #C-41020
PBS buffer w/o Ca^2+^ and Mg^2+^	Animated/Bioconcept	Cat #3–05P29-M
Chemicals, recombinant proteins, and antibodies		
Bovine serum albumin (BSA)		
Ammonium chloride	Sigma-Aldrich	Cat #A9434
Triton X-100	Sigma-Aldrich	
4′,6-Diamidino-2-phenylindole (DAPI)	Sigma-Aldrich	Cat #D9542
Paraformaldehyde (PFA)	Sigma-Aldrich	
α-Adenovirus E1A antibody M58 (mouse)	Thermo Fisher	Cat #MA5-13643
AlexaFluor 488 polyclonal antibody (rabbit, α-mouse)	Thermo Fisher	Cat #A-11094
Experimental models: cell lines		
Human: A549	American Type Culture Collection (ATCC)	ATCC CCL-185
Software and algorithms		
Anaconda Python v3.8.16	Anaconda, Inc.	https://www.anaconda.com/
pandas v1.5.3	Pandas development team (70)	https://pandas.pydata.org/
NumPy v1.24.3	NumPy development team (71)	https://numpy.org/
SciPy v1.10.1	SciPy development team (44)	https://scipy.org/
Other		
Automated high-throughput microscope ImageXpress Micro Confocal (IXM-C)	Molecular Devices	N/A^*a*^
MetaXpress v6.2.3.733	Molecular Devices	N/A

^*a*^ N/A, not applicable.

#### Infection assay and staining

For infection experiments, cell cultures were trypsinized, and cells were re-suspended in the DMEM growth medium. Cells were adjusted to a concentration of 100,000 cells per ml, and 5,000 or 10,000 cells were seeded in 50 or 100 µl medium overnight in 384 or 96 well plates, respectively. On the next day, cells were infected by adding 50 or 100 µl AdV-C5-E1A-FS2A-GFP inoculum, as indicated in the figure legends. At 24 h postinfection, cells were fixed for 30 min by the addition of paraformaldehyde (PFA) to a final concentration of 4%. Subsequently, cells were washed with PBS and quenched by adding 50 µL of 50 mM ammonium chloride in PBS for 10  min, followed by permeabilization with 50 µl 0.25% Triton X-100 in PBS for 5  min. For immunofluorescence staining of wild-type virus infections, cells were blocked with BSA, followed by incubation with mouse α-E1A M58 primary antibody (Thermo Fisher, #MA5-13643) and AlexaFluor 488 α-mouse secondary antibody (Thermo Fisher, #A-11094) for 1 h at room temperature. Finally, cells were stained with DAPI for 15 min.

#### Automated transmitted light and fluorescence microscopy

Transmitted light (TL) and fluorescence images were acquired using the high-throughput microscope ImageXpress Micro Confocal (IXM-C, Molecular Devices). TL images were acquired with a 4× air objective and fluorescence images using a 10× or 20× air objective.

#### Nuclear segmentation and infection index calculation

Image analyses were performed using a custom Python-based pipeline. Nuclei were segmented using the Stardist (72) framework with default parameters. Median GFP fluorescence intensities were determined for each nucleus. The infection threshold was selected at the 99.9th percentile of the uninfected parental populations, and nuclei exceeding this threshold were labeled as infected.

#### Plaque segmentation

Plaque segmentation and quantification were performed using software Plaque 2.0 (52).

### Quantification and statistical analysis

#### Comparison of biological and technical noises

Coefficient of variation (CV) is defined as the ratio of the standard deviation σ and the mean µ of a population as $CV=\frac{\sigma }{\mu }$. Technical noise was assessed by the CVs between the technical replicates, while biological noise was assessed by the CVs between the different clonal populations. Technical $\text{CV}=\frac{1}{n}\sum_{i=1}^{n}\frac{\sigma_{i}}{\mu_{i}}$, and biological $\text{CV}=\frac{\sigma }{\;\frac{1}{n}\sum_{i=1}^{n}\mu_{i}}$, where Σ is the sum operator; n is the number of samples; σ is the standard deviation; μ is the mean; and i refers to the index of a sample. $\sigma_{i}$ refers to the standard deviation of the technical replicates of a given biological sample, while σ refers to the standard deviation across all means of all biological samples. Biological and technical noises were compared using the SciPy implementation of the two-sided, non-parametric Wilcoxon signed-rank test (44).

## Data Availability

Raw image data for the Luria–Delbrück experiments are deposited on BioImage Archive (73) under the accession number S-BIAD1323. The data used for generating the plots are also available in Table S1. Any additional information can be obtained upon request by contacting the corresponding author Prof. Dr. Urs Greber (urs.greber@mls.uzh.ch).

## SUPPLEMENTAL MATERIAL

Fig. S1 msphere.00454-24-s0001.pdf Stability of threshold.

Fig. S2 msphere.00454-24-s0002.pdf Comparison to wild-type AdV-C5, plaque formation at late infection time points, and influence of nuclear area on infection index.

Supplemental Material msphere.00454-24-s0003.pdf Legends for supplemental figure and table.

Table S1 msphere.00454-24-s0004.xlsx Source data for figures.

ASM does not own the copyrights to Supplemental Material that may be linked to, or accessed through, an article. The authors have granted ASM a non-exclusive, world-wide license to publish the Supplemental Material files. Please contact the corresponding author directly for reuse.
