# Supplementary figures and images for: Preexisting cell state rather than stochastic noise confers high or low infection susceptibility of human lung epithelial cells to adenovirus
Source: mSphere. 2024 Sep 24;9(10):e00454-24. doi: 10.1128/msphere.00454-24 (PMC11542551; doi:10.1128/msphere.00454-24)

**A**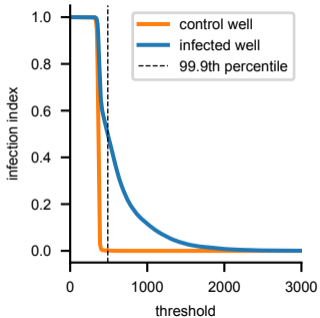**B**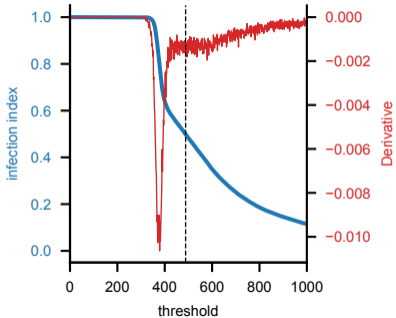

Supplement: Fig. S1 — Stability of threshold. [file msphere.00454-24-s0001.pdf]

**A**

median GFP intensity (AU)

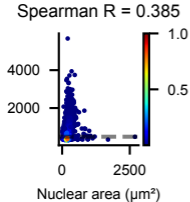**B**number of plaques  
AdV-C5-IX-FS2A-GFP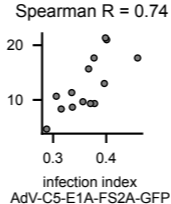**C**infection index  
AdV-C5-E1A-FS2A-GFP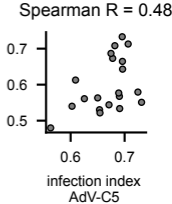

Supplement: Fig. S2 — Comparison to wild-type AdV-C5, plaque formation at late infection time points, and influence of nuclear area on infection index. [file msphere.00454-24-s0002.pdf]
