# Supplementary material for: Preexisting cell state rather than stochastic noise confers high or low infection susceptibility of human lung epithelial cells to adenovirus
Source: mSphere. 2024 Sep 24;9(10):e00454-24. doi: 10.1128/msphere.00454-24 (PMC11542551; doi:10.1128/msphere.00454-24)
Supplement: Supplemental Material — Legends for supplemental figure and table. [file msphere.00454-24-s0003.pdf]

Supplemental Material

'Preexisting cell state rather than stochastic noise confers high or low infection susceptibility of human lung epithelial cells to adenovirus'

Anthony Petkidis, Maarit Suomalainen, Vardan Andriasyan, Abhyudai Singh & Urs F. Greber

Table of contents

SUPPLEMENTAL FIGURE 1: STABILITY OF THRESHOLD 1
SUPPLEMENTAL FIGURE 2: COMPARISON TO WILDTYPE ADV-C5, PLAQUE FORMATION AT LATE INFECTION TIME POINTS, AND INFLUENCE OF NUCLEAR AREA ON INFECTION INDEX 1
SUPPLEMENTAL TABLE 1: SOURCE DATA FOR FIGURES 1-3 AND SUPPLEMENTAL FIGURES 1 AND 2 2

Supplemental Figure 1: Stability of threshold

(A) Infection indices of an uninfected (orange) and infected (blue) well across different potential threshold values. The threshold for the infection cutoff was determined as the 99.9th percentile of the uninfected control population. Under this threshold, the infected well had an infection index of 50 %. **(B)** Infected well (blue) from (A) plotted over the threshold interval from 0 to 1000. The red curve shows the derivative obtained by numerical differentiation. The threshold value is at a position with a stable derivative, indicating that slight variations in the threshold value do not affect the infection index disproportionately.

Supplemental Figure 2: Comparison to wildtype AdV-C5, plaque formation at late infection time points, and influence of nuclear area on infection index

(A) Correlation between nuclear area and median nuclear GFP intensity. Color bar indicates Gaussian kernel density estimate, n = 81,761 (for visual clarity only 1000 data points are shown). **(B)** Correlation between infection indices of AdV-C5-E1A-FS2A-GFP at 1 dpi and number of plaques of AdV-C5-IX-FS2A-GFP at 4 dpi, n = 20. **(C)** Comparison of infection indices of reporter virus (AdV-C5-E1A-FS2A-GFP) and wildtype virus (AdV-C5) for selected

subpopulations, n = 14. Infection indices for the reporter virus were calculated using GFP fluorescence (AdV-C5-E1A-FS2A-GFP), and immunofluorescence staining of E1A in case of AdV-C5.

Supplemental Table 1: Source data for Figures 1-3 and supplemental Figures 1 and 2

The table contains the raw data for Figures 1B, 2A, 2B, 3B, 3C, 3D, 3E, 3F as well as Supplemental Figures 1A, 1B, 2A, 2B, 2C.
